# Nutritional Status of Bariatric Surgery Candidates

**DOI:** 10.3390/nu10010067

**Published:** 2018-01-11

**Authors:** Aliaa Al-Mutawa, Alfred Kojo Anderson, Salman Alsabah, Mohammad Al-Mutawa

**Affiliations:** 1 Department of Food Science and Nutrition, College of Life Sciences, Kuwait University, Kuwait City 13034, Kuwait; round0cube@gmail.com (A.A.-M.); alfanders@gmail.com (A.K.A.); 2 Department of Surgery, College of Medicine, Kuwait University, Kuwait City 13034, Kuwait; 3 Computer Science Department, College of Computing Sciences and Engineering, Kuwait University, Kuwait City 13034, Kuwait; Almutawa@cs.ku.edu.kw

**Keywords:** bariatric surgery, obesity, nutrients

## Abstract

Obesity is a global epidemic affecting populations globally. Bariatric surgery is an effective treatment for morbid obesity, and has increased dramatically. Bariatric surgery candidates frequently have pre-existing nutritional deficiencies that might exacerbate post-surgery. To provide better health care management pre- and post-bariatric surgery, it is imperative to establish the nutritional status of prospective patients before surgery. The aim of this study was to assess and provide baseline data on the nutritional status of bariatric candidates. A retrospective study was conducted on obese patients who underwent bariatric surgery from 2008 to 2015. The medical records of 1538 patients were reviewed for this study. Pre-operatively, the most commonly observed vitamin deficiencies were Vitamin D (76%) and Vitamin B_12_ (16%). Anemia and iron status parameters were low in a considerable number of patients before surgery, as follows: hemoglobin 20%, mean corpuscular volume (MCV) 48%, ferritin 28%, serum iron 51%, and transferrin saturation 60%. Albumin and transferrin were found to be low in 10% and 9% of the patients, respectively, prior to surgery. In addition to deficiencies, a great number of patients had hypervitaminosis pre-operatively. Excess levels of Vitamin B_6_ (24%) was the most remarkable. The findings in this study advocate a close monitoring and tailored supplementation pre- and post-bariatric surgery.

## 1. Introduction

Obesity is associated with low micronutrient levels. Although obese individuals have excess energy stores, they are quite often not well nourished. Many obese subjects have already-existing nutritional deficiencies before bariatric procedures. These deficiencies commonly include iron, Vitamin B_12_, thiamin, folate, and Vitamin D [1,2,3]. Screening and correction of micronutrient deficiencies preoperatively are crucial, as these deficiencies may be more exacerbated following bariatric procedures, leading to devastating conditions.

While several studies have described nutrient status pre- and post-bariatric surgery, there are no publications in the literature that investigate nutritional status pre-surgery among Kuwaiti patients. Moreover, some micronutrient statuses have not been studied extensively. To the best of our knowledge, reports on Vitamin B_2_ status pre-bariatric surgery are scarce. Given the growing practice of bariatric surgery worldwide, and in Kuwait in particular, it would be extremely important to assess the nutritional status prior bariatric surgery to provide an insight into the current nutritional status. The objective of this study was therefore to investigate pre-operative nutritional status among bariatric candidates. This information could assist healthcare teams in getting a better understanding of the present status of the patient, and consequently provide better health care preparation and management pre- and post-bariatric surgery, e.g., through nutritional screening, dietary and supplementation plans, frequency of follow-up in outpatients’ clinics, and regularity of blood testing.

## 2. Methods

### 2.1. Subjects

A retrospective analysis of medical records and bariatric database of 1538 patients who underwent bariatric surgery between October 2008 and September 2015 at Al-Amiri Hospital, Kuwait, was performed. Patients were excluded if they had a history of previous bariatric operations. All ethical protocols were approved by the Ministry of Health, Kuwait.

Pre-operatively, patients were assessed using a multidisciplinary approach to evaluate surgical eligibility. Pre-operative assessments included medical background, anthropometric measurements, nutritional evaluation, abdominal ultrasound, and upper gastrointestinal endoscopy. Nutritional parameters that were evaluated among the bariatric surgery candidates followed in this study were albumin, transferrin, Vitamin B_1_, Vitamin B_2_, Vitamin B_6_, folate, Vitamin B_12_, Vitamin D, Red Blood Cell (RBC), hemoglobin, hematocrit, Mean Corpuscular Volume (MCV), ferritin, iron, and transferrin saturation.

Blood samples were obtained and analyzed according to standard laboratory protocols employed by the hospital for routine analysis, and the reference ranges adopted for this study are shown in Table 1. Nutritional deficiency or excess levels were defined as a concentration below or above the normal reference range of the hospital laboratory.

Ethical approval to conduct the study was obtained from the Ministry of Health and Kuwait Institute for Medical Specialization Ethical Approval Board (Date of approval: 9 February 2016; ethical approcal code 207). Written informed consent was obtained from all participants.

### 2.2. Statistical Analysis

Data were coded and analyzed using SPSS Version 24 (IBM Corp, Armonk, NY, USA), GraphPad Prism Version 7 (GraphPad Software, La Jolla, CA, USA), and Microsoft Excel 2010 (Microsoft Office Professional Plus 2010). Data were tested for normality using Shapiro-Wilk test and Skewness-Kurtosis test. Descriptive statistics—namely, mean ± Standard Deviation (SD) or %—were used as appropriate. Differences between mean values in two groups (Male vs. Female) were analyzed using independent samples *t*-test or Mann-Whitney U test. For all statistical tests, *p* values ≤ 0.05 were considered statistically significant.

## 3. Results

### 3.1. Demographic

The demographic baseline characteristics of the patients studied are shown in Table 2. The study included 1123 (73%) female and 415 (27%) male patients. The great majority of patients were Kuwaitis (89%). The mean age of patients was 35 ± 11.2 years. All patients underwent a laparoscopic sleeve gastrectomy (LSG). Pre-operative mean weight was 123.8 ± 24.3 kg, and body mass index (BMI) was 46.1 ± 8.0 kg/m^2^. This corresponds to an average excess weight of 56.5 ± 21.7 kg before operation.

### 3.2. Protein Status Pre-Bariatric Surgery

#### Albumin and Transferrin

Data for the analyzed nutrients of the bariatric candidates are summarized in Table 1 and Table 3. The average concentration of albumin in the serum specimen of patients was 38.4 ± 3.0 g/L. The average for all male patients was 39.5 ± 3.0 g/L and 38.0 ± 3.0 g/L for females. Overall, 10% of the patients had hypoalbuminemia with concentrations of albumin below the reference range (Table 3).

While the prevalence of high transferrin levels was almost insignificant, the prevalence of low levels was evident, as 9% of the prospective patients had concentrations of transferrin below the reference range. Overall, mean transferrin levels fell within the normal range of 2.15–3.8 g/L (Table 1). Between the genders, females showed higher (2.9 ± 0.5 g/L) transferrin levels than males (2.7 ± 0.6 g/L), but not significantly.

### 3.3. Vitamin Status Pre-Bariatric Surgery

#### 3.3.1. Vitamin B_1_

The prevalence of Vitamin B_1_ deficiency was negligible prior to surgery. The mean level among all patients was 65.0 ± 28.0 ng/mL prior to surgery, and was within the normal range (20–100 ng/mL). Excess cases were more prevalent than deficiencies; pre-operatively, 0.3% of the patients had Vitamin B_1_ deficiency while 7% had excess levels of the vitamin. On average, male patients showed significantly higher levels of Vitamin B_1_ (72.3 ± 35.6 ng/mL) than female patients (62.0 ± 23.5 ng/mL) (*p* = 0.000); still, all were within normal range (Table 1).

#### 3.3.2. Vitamin B_2_

Less than 1% of patients had Vitamin B_2_ deficiency prior to operation, while 16.7% had excess levels (Table 3). On the whole, the mean Vitamin B_2_ level (245.5 ± 80.6 ng/mL) was within the normal range before surgery. However, male patients were more frequently observed with higher Vitamin B_2_ level (258 ± 103.4 ng/mL) than females (241.5 ± 68.7 ng/mL) before surgery.

#### 3.3.3. Vitamin B_6_

Among the population studied, no cases of Vitamin B_6_ deficiency were identified, while there were excess levels in a remarkable 24.1% of patients, pre-operatively. However, on average, the mean level of 28.4 ± 27.2 ng/mL among all patients was within the normal range. On a gender basis, the mean values were 33.9 ± 40.9 ng/mL and 26.1 ± 18.7 ng/mL for males and females, respectively, and were significantly different (*p* = 0.001).

#### 3.3.4. Folate

None of the patients was identified with folate deficiency before surgery, while 3.3% had excess levels. The observed mean serum folate level of 23.0 ± 9.0 mmol/L was within the normal range, and female patients showed higher levels (23.4 ± 9.1 mmol/L) than males (21.8 ± 8.9 mmol/L), although not significantly.

#### 3.3.5. Vitamin B_12_

The prevalence of pre-operative Vitamin B_12_ deficiency was 16.4%, while 0.4% of the patients had excess levels. The mean serum Vitamin B_12_ level was 178.6 ± 91.4 pmol/L, which was within the normal range pre-operatively. Male patients were more frequently observed with higher serum Vitamin B_12_ levels (182.8 ± 93.0 pmol/L) than female patients (176.6 ± 91.2 pmol/L), but the differences were not statistically significant (*p* > 0.05).

#### 3.3.6. Vitamin D

The majority of patients (76%) were Vitamin D deficient before surgery, with a mean level of 37.0 ± 31.0 nmol/L, and 0.2% of the sample patients with excess Vitamin D levels. Overall, female patients were indicative of higher Vitamin D levels (39.6 ± 33.6 nmol/L) than male patients (30.0 ± 21.6 nmol/L), both of which were below the preference range.

### 3.4. Anemia and Iron Status Pre Bariatric Surgery

#### 3.4.1. Red Blood Cells (RBC)

Before surgery, only 3.1% of the patients were found to have low RBC levels. Mean RBC levels was observed to be 4.9 ± 0.5 × 10^12^/L among all patients. The mean for female patients was 4.8 ± 0.5 × 10^12^/L and for male patients it was 5.2 ± 0.6 × 10^12^/L, both of which were within their respective normal ranges.

#### 3.4.2. Hemoglobin

Anemia status was assessed based on hemoglobin levels. The mean hemoglobin levels in 1079 patients before surgery were 133.4 ± 15.3 g/L, which was within the normal range. However, the data indicated that 19.6% of the patients had low hemoglobin levels, and on a gender basis, 14.4% male and 21.6% females had low hemoglobin levels prior to surgery. Male patients on average had significantly higher levels (145.4 ± 15.2 g/L) than females (129.0 ± 12.7 g/L) (*p* = 0.000).

#### 3.4.3. Hematocrit

Besides using hemoglobin as a parameter, anemia was also assessed by hematocrit levels. Before surgery, the mean level was found to be 0.40 ± 0.04 L/L, while on gender basis, the means were 0.43 ± 0.04 L/L and 0.39 ± 0.03 L/L for males and females, respectively. All values were within the normal range. However, 18.4% of both genders indicated low hematocrit levels, while 17.8% males and 18.6% females indicated low hematocrit levels.

#### 3.4.4. Mean Corpuscular Volume (MCV)

Before surgery, 47.9% of the patients had microcytosis while no incidence of macrocytosis was detected. The pre-operative MCV mean value was 82.1 ± 6.8 fL, indicating microcytosis (Table 1). Between genders, male patients had significantly higher MCV mean value (83.6 ± 6.4 fL) in comparison to female patients (81.5 ± 6.9 fL) (*p* = 0.000). Thus, while male patients were within the normocytic level, female patients showed microcytic levels pre-operatively.

#### 3.4.5. Ferritin

Low ferritin levels were noted in 28.2% of the patients before surgery, while there was no record of excess ferritin in any of the patients (Table 3). The mean value for all patients was 46.0 ± 65.1 ng/mL, and males had a significantly higher mean than females (*p* = 0.000) at 123.2 ± 108.5 ng/mL and 29.4 ± 34.8 ng/mL, respectively. Overall, mean ferritin levels were well within the normal range before surgery (Table 1).

#### 3.4.6. Iron

The mean serum iron level in all patients was 11.9 ± 6.5 µmol/L, which was within the normal range. However, low serum iron levels were observed in almost half of the patients (50.5%) pre-operatively (Table 3), with only 1% showing excess. Overall, mean serum iron levels were almost within the normal range before surgery, and both males and females had almost the same level of serum iron, 12.0 ± 4.7 µmol/L and 11.9 ± 6.9 µmol/L, respectively.

#### 3.4.7. Transferrin Saturation

The mean transferrin saturation determined was 18.2 ± 11.3%. A great number of patients (59%) were found with low transferrin saturation levels (<20%) (Table 3), which implies a high prevalence of iron deficiency. On a gender basis, males had a higher value of 23.3 ± 15.8%, which was within the normal range, while females were significantly deficient (16.3 ± 8.6%).

## 4. Discussion

### 4.1. Nutritional Deficiencies Pre Bariatric Surgery

Several studies have examined nutritional deficiencies among morbidly obese patients prior to bariatric procedures. Schweiger et al. [4] studied nutritional deficiencies in 114 bariatric candidates who underwent surgery between 2006 and 2008. The prevalence of pre-operative nutritional deficiencies was 35% for iron, 24% for folate, 24% for ferritin, 3.6% for Vitamin B_12_, 2% for phosphorous, and 0.9% for calcium. Hemoglobin and Mean Corpuscular Volume (MCV) levels were low in 19% of the patients. High levels of Parathyroid Hormone (PTH) were found among 39% of the patients. No hypoalbuminemia was encountered. Low iron and ferritin were more common in females than males (40.8 vs. 14.3%) and (31.8 vs. 0%), respectively. Similarly, another study conducted by Al-Mulhim [5] prospectively evaluated nutritional status in 112 patients. Pre-operatively, 64% of the patients had one deficiency, and 11% had more than one. Deficiencies rates were reported as follows: hemoglobin 24%, iron 11.6%, Vitamin D 60%, Vitamin B_12_ 1.8%, and folate 0.9%.

According the latest report from the 2017 International Federation of Surgical Obesity global registry Kuwait (100.0%), Australia (100.0%) and Saudi Arabia (100.0%) submitted and reported the highest rates of sleeve gastrectomy operations [6]. This data illustrates the importance of investigating the nutritional effects of LSG on bariatric patients in Kuwait. Data from this study showed that bariatric surgery candidates had pre-existing nutritional deficiencies, which included Vitamin D, iron, Vitamin B_12_, and protein. Similar conditions of micronutrient deficiencies have been found in morbidly obese patients prior sleeve gastrectomy [7]. The importance of pre-assessment of the nutritional status of bariatric surgery candidates has also been reported previously [4,8]. Nutritional deficiencies among bariatric surgery candidates are commonly attributed to unhealthy dietary and lifestyle habits [9,10], including consumption of non-varied, high-calorie and high-fat diets. Obese individuals often displace nutritious foods with high-calorie foods that are rich in refined carbohydrates and fat. Moreover, chronic dieting, which is common among obese individuals, might further deteriorate their nutritional status as a result of food restrictions. Aside from diet and lifestyle, a further explanation involves the volumetric dilution factor. Obese individuals have relatively high amounts of total body water, and their extracellular compartment appears to be more expanded than the intracellular compartment [11]. Aasheim et al. [10] suggested that low micronutrient levels might be related to the dilution effect of the extracellular fluid on micronutrient concentrations.

### 4.2. Protein Deficiency Pre Bariatric Surgery: Albumin and Transferrin

Albumin and transferrin levels were used as potential indicators for protein status, and were low in about 10% of the studied patients pre-operatively. This might indicate that the great majority (~90%) of the bariatric candidates had sufficient protein intake. This finding is in line with the reported protein intake in the general population of Kuwait. According to the national nutrition survey in Kuwait, the estimated protein intake was 110 g/day in males and 67 g/day in females [12]. These correspond to a 99.6% and 98.4% of males and females, respectively, who met the Acceptable Macronutrient Distribution Range (AMDR).

Reports of albumin status vary widely in the literature, with the percentage of low albumin ranging from 0 to 27% before bariatric surgery [4,7,13,14,15]. It is important to note that albumin and transferrin levels do not always reflect actual protein status, since they are affected by other conditions, including inflammation, liver disease, and nephrotic syndrome, which were not investigated in this study [16]. Since obesity is associated with chronic low-grade inflammation, acute phase protein levels, including albumin and transferrin, might be altered [17]. Hence, the observed low levels of albumin and transferrin in the current study should be carefully interpreted, as they may not necessarily indicate insufficient protein intake.

### 4.3. Vitamin Deficiencies Pre Bariatric Surgery

#### 4.3.1. B Vitamin Deficiencies Pre Bariatric Surgery

Vitamin B_12_ was the only B vitamin that showed considerable deficiency prevalence (16.4%) prior to surgery (Table 3). Vitamin B_12_ deficiency findings were in line with earlier studies (13–16%) [7,18,19], nevertheless, B_12_ deficiency proved to be lower in the general population of Kuwait, with 1.3% of males and 5.6% of females showing deficiencies [20]. However, several studies have also reported significant deficiencies in Vitamin B_1_, B_6_, and folate, which contradict the current findings [4,7,13,14,18]. These variations in B Vitamin statuses may be partially explained by the differences in the extent of food fortifications between countries. It could also be related to food and supplement intake, which was not assessed in this study. According to the national nutrition survey in Kuwait, the mean intake of Vitamins B_1_, B_2_, B_6_ and B_12_ was greater than the Estimated Average Requirement (EAR) values in both genders [12]. Data on folate showed that more than two-thirds of the Kuwaiti population did not meet the EAR [12]. Overall, these findings are comparable to the current findings as negligible deficiency prevalence was observed in Vitamin B_1_, B_2_, and B_6_. A third possible explanation for the differences between the current data and some literature is the prevailing cultural factor that forbids alcohol consumption. Chronic alcoholism can be a contributing factor to Vitamin B_1_ and folate deficiencies, as alcohol interferes with the active transport of Vitamin B_1_ and folate across the intestinal wall and hastens their excretion in urine [16,21,22]. Accordingly, this might justify the negligible prevalence of Vitamin B_1_ and folate deficiencies before surgery in comparison to previous studies.

#### 4.3.2. Vitamin D Deficiency Pre Bariatric Surgery

Vitamin D deficiency was the most prevalent before surgery, affecting about 76% of the surgery candidates (Table 3). This high prevalence of Vitamin D deficiency is consistent with previously reported range of 60–91% [5,7,13,14,15]. Regarding the general population in Kuwait, data from Kuwait National Nutritional Survey (KNNS) showed a high prevalence of Vitamin D deficiency among Kuwaiti adults [22]. According to a cross-sectional study of 960 adults enrolled for the KNNS, approximately 56% of the Kuwaiti adults had Vitamin D inadequacy (25 (OH) D = 12–19.9 ng/mL), and 27% had Vitamin D deficiency (25 (OH) D < 12 ng/mL) [23]. Molla et al. [24] also assessed Vitamin D levels in Kuwaiti mothers and their neonates in a total of 128 mother–neonate pairs. Data showed that 40% of the mothers and 60% of the neonates are Vitamin D deficient on the day of delivery. The high prevalence of Vitamin D deficiency found in the current study is also comparable to other findings from Middle Eastern countries [25]. According to the cross-sectional study involving 834 healthy Saudi Arabian men by Ardawi et al. [26], Vitamin D deficiency was present in 87.8% of the participants.

Vitamin D deficiency in the current bariatric candidates can be attributed to several causes. One reason is the decreased dietary consumption of Vitamin D-rich sources, including fortified dairy products. A second reason is the reduced exposure to sunlight due to limited outdoor activities, clothing habits, and use of sunscreen. The psychological status of obese individuals and the cultural and lifestyle factors of the population might further explain the limited sun exposure. A third possible reason for Vitamin D deficiency is the sequestration of Vitamin D in adipose tissues. The degree of adiposity appears to be inversely correlated with Vitamin D levels. Correspondingly, several studies have reported that obese individuals tend to have lower levels of Vitamin D due to its increased uptake in adipose tissue [27,28,29]. A fourth reason for deficiency might be related to the decreased synthesis of Vitamin D by the liver as a result of impaired liver function due to fatty liver disease, which is common among obese individuals [30]. Lastly, regarding the variation in Vitamin D deficiency prevalence in the literature, it could also be related to the geographical, seasonal, and fortification policy differences.

### 4.4. Anemia and Iron Deficiency Pre Bariatric Surgery

Using low hemoglobin levels as an indicator for anemia, anemia was observed in about 20% of the bariatric candidates in this study, which supports previously reported values of 18–24% [4,5,9,15]. However, anemia has been variably reported in the literature, and others have observed a much lower prevalence (1–5%) [7,13,19]. The prevalence of anemia according to hemoglobin status was shown to be at a lower level in the general population of Kuwait as compared to bariatric candidates, showing deficiencies in 2.5% in males and 17.1% in females aged 20–49 [20]. Iron, folate, and B_12_ deficiencies are the main contributors to the development of anemia. However, given the low level of iron status parameters in our study population, it can be stated that the observed anemia was mostly due to iron deficiency. Iron biochemical parameters such as ferritin, serum iron, and transferrin saturation indicated poor iron status pre-operatively. Low ferritin was found in 28% of our patients, which is in good agreement with the 24% reported by Schweiger et al. [4]. Low serum iron was observed in half of the patients before surgery, which matches the data of Ben-Porat et al. [18] (47%). On the contrary other previous studies have reported a much lower prevalence in terms of low ferritin (1–10%) [7,9,18,31] and serum iron levels (7–29%) [5,15,31,32].

In terms of the prevalence of anemia and iron deficiency in the general Kuwaiti population, data from a cross-sectional study with a sample size of 1830 showed that the prevalence of anemia in adults was 3% in males and 17% in females [20]. Similarly, the prevalence of iron deficiency based on low serum ferritin levels was 4% in males and 19% in females [20]. In comparison to the general population, the prevalence of anemia and iron deficiency appeared to be higher in our obese sample, and was more noticeable in the male gender.

The high percentages of pre-operative anemia and iron deficiency may be attributed to inadequate iron intake due to poor dietary choices. A second cause of deficiency may include poor adherence to oral iron supplementation by anemic patients due to its gastrointestinal side effects. A third cause of deficiency could be the predominance of the female gender (73%) in the reproductive age in our sample. Women of reproductive age are at increased risk of iron deficiency anemia due to blood loss through menstruation. Furthermore, blood investigation of ferritin, serum iron, and transferrin saturation were not part of the routine preoperative assessment; hence, these tests may only be requested when deficiencies were suspected.

### 4.5. Excess Micronutrient Levels Pre Bariatric Surgery

In addition to deficiency findings, some patients were found to have excess micronutrient levels, which is consistent with data reported by van Rutte et al. [7]. These excess levels might be due to the consumption of large doses of over-the-counter supplements by the patient’s own initiative, or by intense preoperative nutritional optimization from healthcare providers.

Excess levels were seen for all the investigated vitamins; however, Vitamin B_6_ was the most remarkable. Vitamin B_6_ was found in excess levels among 24.1% of the bariatric surgery candidates. This data is similar to a previous report of Vitamin B_6_ hypervitaminosis being found in 21% of pre-operative patients [7]. Regarding the mean levels, male patients in the current study showed excess Vitamin B_6_ with average plasma concentrations of 33.9 ng/mL, compared to the normal range of 7–30 ng/mL. Excessive increase in Vitamin B_6_ levels might lead to sensory neuropathy with gait changes, peripheral sensation, and lack of muscle coordination [22]. Like other B vitamins, Vitamin B_6_ is water soluble, and thus is easily eliminated in urine. Therefore, toxicity due to B_6_ is unlikely at reasonable daily doses, but it has been reported that regular consumption of as little as 200 mg could cause adverse effects [33]. As for folate toxicity, it is considered low. However, high intake of folate from supplements may obscure signs of Vitamin B_12_ deficiency [22]. If Vitamin B_12_ deficiency is left untreated, serious neurological damage may occur. Regarding Vitamin B_1_, some reports have described toxicity symptoms including anxiety, pruritus, respiratory distress, nausea, abdominal pain, and shock [22]. It is important to note that neither supplement intake nor clinical manifestations of vitamins toxicity have been investigated in the present study. Consequently, it is hard to know whether the observed excess levels are of any clinical relevance.

## 5. Conclusions

The rising prevalence of obesity is causing a parallel increase in the use of bariatric surgery. Nutritional status is one of the main concerns in the bariatric field. Since nutritional deficiencies are common among obese individuals, nutritional assessment and optimization before bariatric surgery is crucial to avoiding further deterioration post surgery. In this study, nutritional deficiencies were found to exist in patients prior to bariatric surgery. Vitamin D, Vitamin B_12_, and iron deficiencies were the most commonly observed before surgery, which suggests more consideration should be given to these micronutrients. Female patients of childbearing age deserve particular attention in term of anemia and iron status. On the other hand, data regarding Vitamin B_2_ do not suggest a need for intense supplementation and monitoring. The current data highlights the importance of pre-operative nutritional screening and optimization. It is important to note that nutritional supplementation should be tailored to patient laboratory tests to prevent excessive increases in micronutrient levels.

## Figures and Tables

**Table 1:** Nutrient status of patients pre bariatric surgery.

Nutritional Parameter	Number of Patients		Mean By Gender			Reference Range
		Sample Mean ± SD	Male Mean ± SD (*n*)	Female Mean ± SD (*n*)	*p*	
**Albumin**	1065	38.4 ± 3.0	39.5 ± 3.0 (288)	38.0 ± 3.0 (777)	0.000 *	35–48 g/L
**Transferrin**	97	2.8 ± 0.5	2.7 ± 0.6 (27)	2.9 ± 0.5 (70)	0.120	2.15–3.8 g/L
**Vitamin B_1_**	637	65.0 ± 28.0	72.3 ± 35.6 (187)	62.0 ± 23.5 (450)	0.000 *	20–100 ng/mL
**Vitamin B_2_**	420	245.5 ± 80.6	258.0 ± 103.4 (120)	341.5 ± 68.7 (300)	0.058	75–300 ng/mL
**Vitamin B_6_**	630	28.4 ± 27.2	33.9 ± 40.9 (183)	26.1 ± 18.7 (447)	0.001 *	7–30 ng/mL
**Folate**	306	23.0 ± 9.0	21.8 ± 8.9 (84)	23.4 ± 9.1 (222)	0.153	7–45 nmol/L
**Vitamin B_12_**	563	178.6 ± 91.4	182.8 ± 92.0 (165)	176.6 ± 91.2 (398)	0.464	<107 pmol/L Deficient; 107–133 pmol/L Indeterminate; 133–675 pmol/L Normal
**25 (OH) Vitamin D**	610	37.0 ± 31.0	30.0 ± 21.6 (167)	39.6 ± 33.6 (443)	0.001 *	<50 nmol/L Deficient; 50–75 nmol/L Insufficient; >75 nmol/L Sufficient; >250 nmol/L Critical High
**RBC**	1079	4.9 ± 0.5	5.2 ± 0.6 (292)	4.8 ± 0.5 (787)	0.000 *	Male: 4.5–5.5 (10^12^/L); Female: 3.8–4.8 (10^12^/L)
**Hemoglobin**	1079	133.4 ± 15.3	145.4 ± 15.2 (292)	129.0 ± 12.7 (787)	0.000 *	Male: 130–170 g/L; Female: 120–150 g/L
**Hematocrit**	1079	0.40 ± 0.04	0.43 ± 0.04 (292)	0.39 ± 0.03 (787)	0.000 *	Male: 0.4–0.5 L/L; Female: 0.36–0.46 L/L
**MCV**	1079	82.1 ± 6.8	83.6 ± 6.4 (292)	81.5 ± 6.9 (787)	0.000 *	<83 fL Microcytic; 83–101 fL Normocytic; >101 fL Macrocytic
**Ferritin**	78	46.0 ± 65.1	123.2 ± 108.5 (14)	29.4 ± 34.8 (64)	0.000 *	Male: 23.9–336.2 ng/mL; Female: 11–306.8 ng/mL
**Iron**	102	11.9 ± 6.5	12.0 ± 4.7 (22)	11.9 ± 6.9 (80)	0.939	11–28 µmol/L
**Transferrin Saturation**	97	18.2 ± 11.3	23.3 ± 15.8 (27)	16.3 ± 8.6 (70)	0.006 *	20−40%

* Significant difference between genders (*p* ≤ 0.05). RBC: Red Blood Cell; MCV: mean corpuscular volume.

**Table 2:** Study demographics.

Demographics	Mean ± SD
**Age (years)**	35 ± 11.2
**Height (cm)**	163.8 ± 9.0
**Weight (kg)**	123.8 ± 24.3
**Body mass index (BMI) (kg/m^2^)**	46.1 ± 8.0
**EW (kg)**	56.5 ± 21.7

**Table 3:** Prevalence of deficient and excess nutrient levels pre bariatric surgery.

Nutritional Parameter	Number of Patients	% Deficient/Low	% Excess
**Albumin**	1065	10.1	0
**Transferrin**	97	9.0	3.0
**Vitamin B_1_**	637	0.3	7.0
**Vitamin B_2_**	420	0.7	16.7
**Vitamin B_6_**	630	0	24.1
**Folate**	306	0	3.3
**Vitamin B_12_**	563	16.4	0.4
**25 (OH) Vitamin D**	610	75.6	0.2
**RBC**	1079	3.1	0
**Hemoglobin**			
Both Genders	1079	19.6	0
Male	292	14.4	
Female	787	21.6	
**Hematocrit**			
Both Genders	1079	18.4	0
Male	292	17.8	
Female	787	18.6	
**MCV**	1079	47.9	0
**Ferritin**			
Both Genders	78	28.2	0
Male	14	21.4	
Female	64	29.7	
**Iron**	102	50.5	1.0
**Transferrin Saturation**	97	59.0	2.0
